# Direct Imaging of Atomic Permeation Through a Vacancy Defect in the Carbon Lattice

**DOI:** 10.1002/anie.202010630

**Published:** 2020-10-25

**Authors:** Kecheng Cao, Stephen T. Skowron, Craig T. Stoppiello, Johannes Biskupek, Andrei N. Khlobystov, Ute Kaiser

**Affiliations:** ^1^ Ulm University Electron Microscopy of Materials Science Central Facility for Electron Microscopy Albert-Einstein-Allee 11 Ulm 89081 Germany; ^2^ University of Nottingham School of Chemistry University Park Nottingham NG7 2RD UK; ^3^ University of Nottingham Nanoscale & Microscale Research Centre (nmRC) University Park Nottingham NG7 2RD UK

**Keywords:** carbon nanotube, graphene, permeation, selective membrane, transmission electron microscopy

## Abstract

Porous graphene has shown promise as a new generation of selective membrane for sieving atoms, ions and molecules. However, the atomistic mechanisms of permeation through defects in the graphenic lattice are still unclear and remain unobserved in action, at the atomic level. Here, the direct observation of palladium atoms from a nanoparticle passing through a defect in a single‐walled carbon nanotube one‐by‐one has been achieved with atomic resolution in real time, revealing key stages of the atomic permeation. Bonding between the moving atom and dangling bonds around the orifice, immediately before and after passing through the subnano‐pore, plays an important role in the process. Curvature of the graphenic lattice crucially defines the direction of permeation from concave to convex side due to a difference in metal‐carbon bonding at the curved surfaces as confirmed by density functional theory calculations, demonstrating the potential of porous carbon nanotubes for atom sieving.

## Graphical abstract

This work reveals the mechanism of atomic permeation through a subnano‐pore in graphenic lattice by in situ aberration‐corrected high‐resolution transmission electron microscopy imaging, highlighting the importance of chemical bonding between the mobile atom and dangling bonds around the subnano‐pore. This new phenomenon and permeation mechanism are likely to play a role in the filtration processes by porous graphenic carbon based membranes.

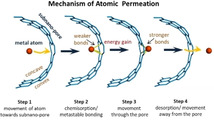

Permeation and transport of atoms, molecules or ions across membranes is one of the most fundamental phenomena in natural and manmade materials. Graphene being an ultimate membrane with nano‐ and sub‐nanometer vacancy defects has been successfully utilized for transport of ions, atoms, molecules in effective separation.^[1]^ Theoretical calculations predicted that single‐layer graphene with sub‐nanometer defects have the ability to filter NaCl salt from water^[2]^ and to separate gases.^[3]^ Indeed, experimental results demonstrate that pristine graphene is impermeable for all the ions, atoms and molecules except H^+^,^[4]^ while porous single‐layer graphene exhibits superb performance for water desalination and purification,^[5]^ molecular sieving^[6]^ with high selectivity and flux. The driving forces for these applications include pressure,[^6a^, ^7^] electric fields[^5^, ^8^] and osmosis^[9]^ across porous graphene. The size and termination of the pore in graphene could influence the selectivity of filtration owing to steric effects and electrostatic interactions.[^2^, ^6b^, ^10^] In addition, cobalt and gold atoms and clusters are able to migrate through the graphitic layers of carbon onions under high energy electron beam irradiation and high temperature, directly observed by transmission electron microscopy (TEM).^[11]^ However, the atomistic mechanisms of permeation are still unknown and they remain unobserved in action, at the atomic level. Carbon nanotubes, graphene sheets rolled up into cylinders, have been applied for mass transfer of gases,^[12]^ liquids,^[13]^ nanoparticles^[14]^ and biomolecules.^[15]^ Analogous to graphene, sub‐nanometer defects may pre‐exist in the lattice of carbon nanotubes^[16]^ or can be created by chemical and physical techniques such as treatment with an oxidant or microwaves.^[17]^ Importantly, in the context of metals, theoretical calculations predicted that a transition metal atom bonding to a sub‐nano vacancy defect on a single‐walled carbon nanotube (SWNT) externally is more stable than when it bonds to the defect internally,^[18]^ which in principle may provide a mechanism for metal atom transport across a defect in SWNT, without any external field gradient.

In this work, we present the direct observation of metal atoms spontaneously penetrating a sub‐nanometer pore in the carbon lattice of nanotube by in situ aberration‐corrected high‐resolution TEM (AC‐HRTEM). We demonstrate with atomic resolution that palladium atoms are able to climb out from the interior of the SWNT and escape into vacuum atom‐by‐atom, detaching from a nanoparticle. Our results show the atomic dynamics and mechanisms of metal permeation through carbon lattice in action, emphasizing the importance of the carbon lattice curvature as a driving factor for atomic permeation.

Palladium nanoparticles were prepared inside SWNT by inserting palladium hexafluoroacetylacetonate complex into SWNT from gas phase followed by thermal decomposition and formation of metallic nanoparticles inside the nanotube.^[19]^ In the initial state the Pd nanoparticle had a length of 5.45 nm and diameter of 0.72 nm controlled by the diameter of SWNT (Figure 1 a). This Pd nanoparticle can be divided into three parts including a crystalline Part I with a (1,1,1) face and 2.48 nm in length, a different crystalline Part II with a (1,0,0) face and 1.25 nm in length, and an amorphous Part III with a length of 1.72 nm. Part III binds to one of the four vacancy defects in SWNT carbon lattice as indicated by red arrows in Figure 1 a. The structural model of Pd nanoparticle@SWNT (Figure 1 b) represents the nanoparticle consisting of 140 Pd atoms confined within a (13,7) SWNT and connected to a defect in the carbon lattice (Figure. S1). The TEM image simulated for this model (Figure 1 c) demonstrates a good fit with the experimental image, and a top view of the structural model illustrates how a vacancy defect may bond to the Pd atoms (Figure 1 d). Both ends of the host SWNT of this Pd nanoparticle are blocked by two other Pd nanoparticles during the whole process, which means the Pd atoms are not able to diffuse in the cavity and then escape from the host SWNT (Figure S2). Time‐series AC‐HRTEM images of Pd nanoparticle@SWNT reveal that stimulated by the 80 keV electron beam at the dose rate of 4.3×10^6^ e^−^ nm^−2^ s^−1^, the Pd atoms in the Pd nanoparticle successively diffuse through a sub‐nanometer defect from inside to outside the SWNT and escape into vacuum, atom‐by‐atom (Figure 2, Movie S1). The changing length of the Pd nanoparticle, estimated total number of metal atoms remaining in the nanotube and in the crystalline Part I of the nanoparticle are plotted as a function of time in Figure 3 a. The number of atoms in the nanoparticle is considered to be proportional to its length, which is a reasonable approximation due to the uniform cylindrical shape of the nanoparticle. From 0 s to 32 s, the Pd nanoparticle moves translationally inside SWNT within a short distance, with the length showing only minor fluctuations caused by the slight re‐arrangement of atoms (Figure 3 a). In addition, a single Pd atom can be observed adsorbed on the defect outside SWNT, as indicated by the black arrow in Figure 3 b, which desorbs into the vacuum at 33 s. After that the Pd atoms began permeating across the defect and escaping into vacuum successively until 61 s. The nanoparticle length becomes reduced by 2.36 nm during this stage as can be described by the linear function (R^2^=0.904

(1) l=-0.077t+8.05(37≤t≤61s)

**Figure 1:**
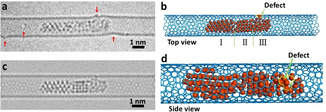
Atomic structure of the Pd nanoparticle entrapped in a host SWNT. a) AC‐HRTEM image of the frame of 11 s in Movie S1 showing a long Pd nanoparticle within a SWNT with several vacancy defects. The defect sites in the carbon lattice are indicated by red arrows. b) Structural model of the Pd nanoparticle@SWNT in (a). The Pd nanoparticle consists of three parts including two different crystalline parts with (1,1,1) face (Part I) and (1,0,0) face (Part II) and an amorphous part (Part III) bonded to a vacancy defect as indicated by a green arrow. c) Simulated TEM image from structural model in (b). d) Enlarged side view of structural model in (b) highlighting the sub‐nano vacancy defect in the carbon lattice colored yellow and indicated by a green arrow.

**Figure 2:**
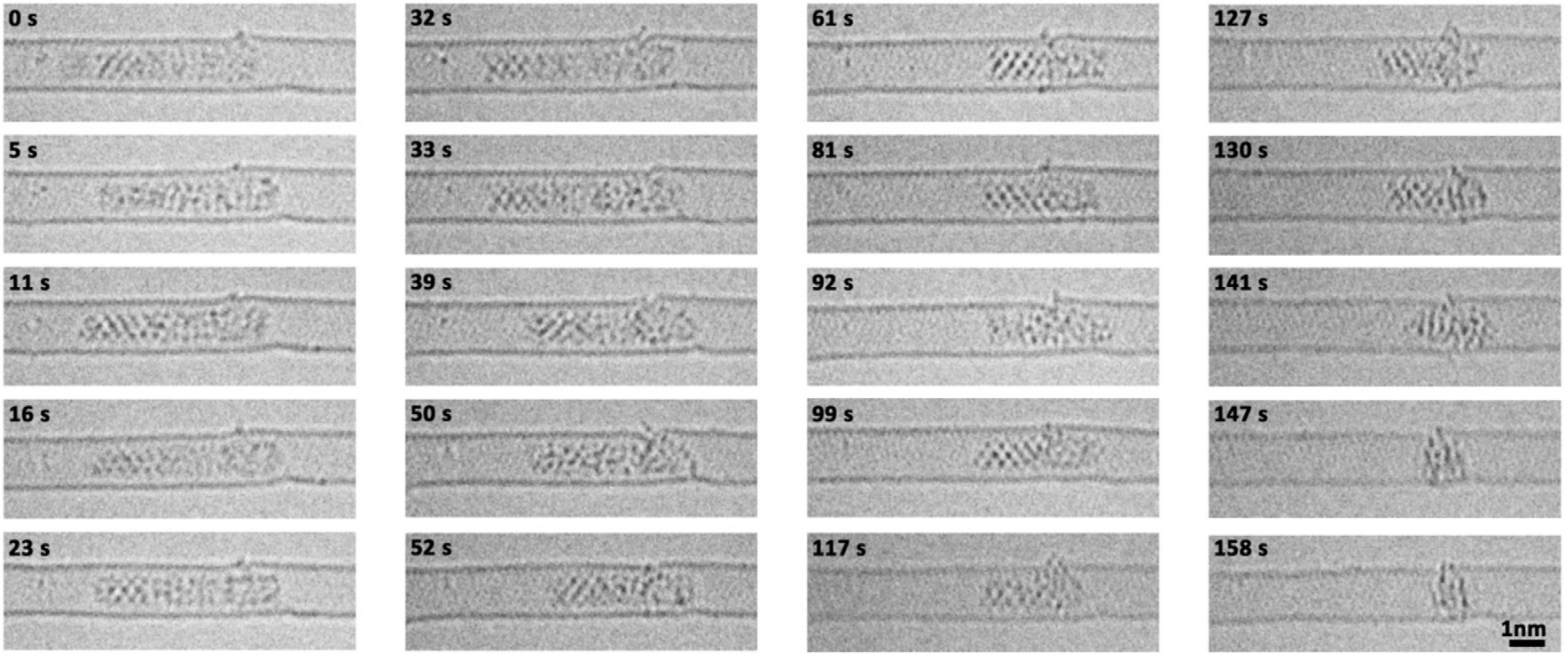
Metal atoms of the Pd nanoparticle permeate across a vacancy defect in the carbon lattice of the host SWNT. Time‐series AC‐HRTEM images from Movie S1 showing the atoms of a Pd nanoparticle permeating across a sub‐nanometer defect under 80 keV electron beam irradiation with an exposure time of 0.5 s.

**Figure 3:**
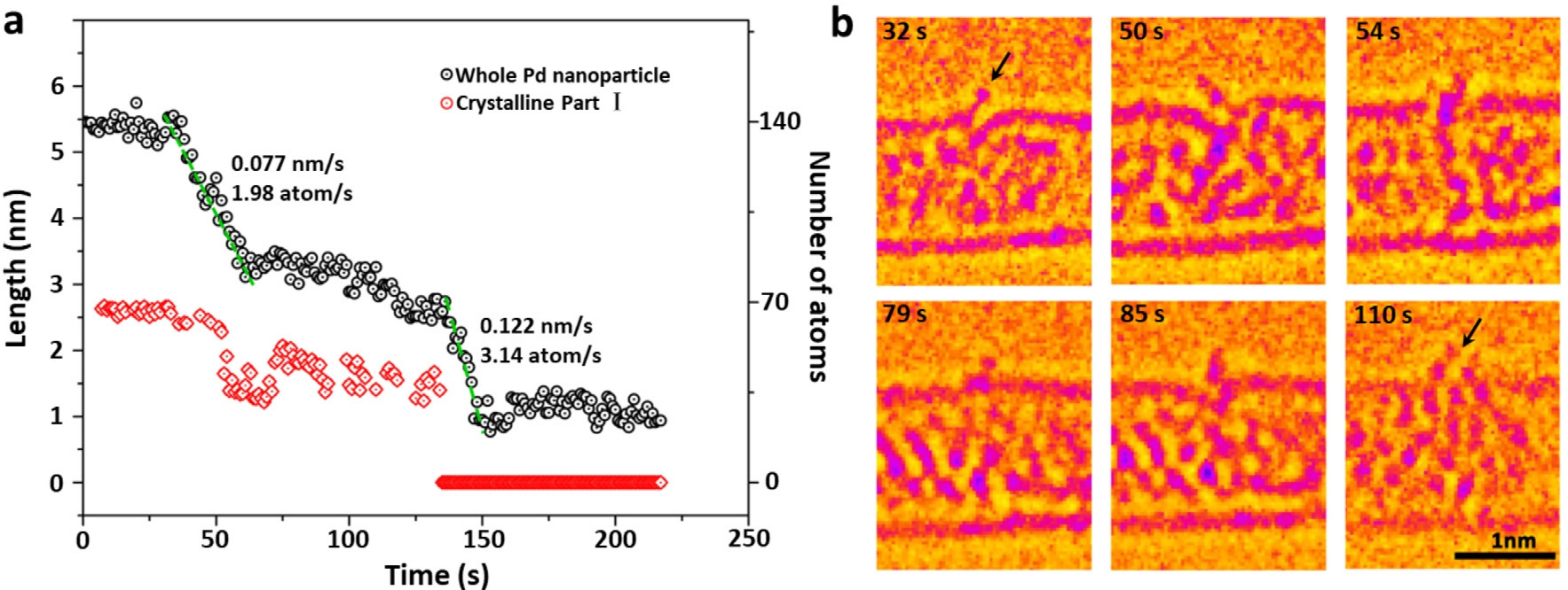
Quantification and highlighted detail of the permeation of the Pd atoms. a) Plot showing the changing length and corresponding number of atoms for the whole Pd nanoparticle (black dots) and the crystalline Part I as a function of time during the permeation process. b) Typical AC‐HRTEM images showing snapshots of the Pd atoms transiently adsorbed on the nanotube outer surface after permeating through the defect (indicated by black arrows).

where *t* is time, indicating a rate of permeation of 1.98 Pd atoms per second. The crystalline Part I remains relatively unchanged under the 80 keV electron beam during the first stage, fluctuating only from 50 s to 60 s, while the crystalline Part II reduced linearly up to 61 s. In addition, the size of the amorphous part connected to the defect remained constant, which indicates that during the first stage the crystalline Part II became disordered into an amorphous state, while the Pd atoms from the amorphous part permeated across the defect. From 62 s to 136 s, the permeation rate significantly reduced, during which time the crystalline Part I was still generally stable with a recognizable crystal face and a constant length around 1.5 nm.

The next fast permeation process started at 137 s which can be described by another linear function (R^2^=0.897

(2) l=-0.122t+19.25(137≤t≤151s)

indicating a higher rate of permeation of 3.14 Pd atoms per second. During this stage, the crystalline Part I significantly amorphized allowing faster permeation. By the end of this stage most of the Pd atoms had left the nanotube and escaped through the defect into the vacuum, and only a small amorphous Pd cluster with diameter of ≈1.0 nm remained. Finally, the remnant Pd cluster started bonding with the host SWNT instead of permeating, which may be caused by the increased surface energy of such a small Pd nanocluster (Movie S1).^[19]^ The residence time of each Pd atom adsorbed on the defect outside of SWNT, before desorbing irreversibly into the vacuum, is long enough for it to be captured and observed by TEM under our experimental conditions (Figure 3 b from Movie S1). In the frames of 32 s and 54 s, a single external Pd atom can be observed while in the other four frames two (50 s) or more Pd atoms (79 s, 85 s, 110 s) were found adsorbed outside of nanotube. These external Pd atoms were sputtered by electron beam immediately with a residence time of less than a second (Figure 2). More detailed permeation processes of Pd atoms are presented in Figure S3. Thus, we directly observed the atomic dynamics of the permeation process through a defect in carbon lattice, in real time and at the atomic level.

Typically, a gradient of pressure, electric field or concentration is required to drive the permeation process.[^5^, ^6^, ^7^, ^8^, ^9^, ^20^] The sole stimulus in our experiments is the 80 keV e‐beam, with no gradient across the carbon lattice. As the cohesive energy (*E_s_*) for Pd bulk metallic crystal is 3.89 eV per atom (Figure 4 a), the barrier for removal of Pd atom from a Pd crystal surface should be expected to be >15 eV (i.e. typically about four times of the cohesive/sublimation energy) considering the coordination number of Pd atoms on the surface is eight on average.^[21]^ Our results clearly show that amorphization of Pd nanoparticle is a prerequisite for the permeation (Figures 2 and 4 a). The amorphous Pd cluster has higher free energy per atom than Pd crystal which can be simply estimated as the latent heat of fusion for Pd metal (*E_f_*=0.18 eV per atom). In addition, the flexible nature of intermetallic bonding within the nanoparticle makes it metastable under the electron beam which could even form dynamical metal atoms with coordination number of one as demonstrated by our previous works.[^19^, ^22^] Thus, the threshold energy of sputtering (*E_d_*) for Pd nanocluster should be expected around 2.0 eV–14.84 eV depending on the coordination number for the Pd atoms on the surface. Considering that the Pd@SWNT in our experiment is an electrically conductive system, the effect of ionization caused by inelastic interactions with the incident electrons is negligible. The most important source of energy is the kinetic energy transferred from the incident electrons to the Pd atoms by elastic scattering with the maximum of 1.78 eV (at the scattering angle of 180°) under our experimental conditions which is not sufficient to directly displace and sputter a Pd atom from a Pd nanoparticle. Indeed, no direct sputtering of Pd atoms from nanoparticles can take place under 80 keV e‐beam as we demonstrated earlier.^[19]^ However, inside the nanotube the Pd nanoparticle clearly loses Pd atoms and emits them through a sub‐nanometer defect (Figure 2), indicating that the contact between the metal and dangling bonds of the defect in the carbon lattice is an essential factor enabling atomic permeation and sputtering. In addition, with the loss of Pd atoms of Part III by permeation, the size of amorphous cluster decreases while the free energy per atom increases, which will also increase free energy of the crystalline atoms of the Part II on the crystalline‐amorphous interface. Thus these crystalline atoms can be amorphized by electron beam irradiation and join into Part III.

**Figure 4:**
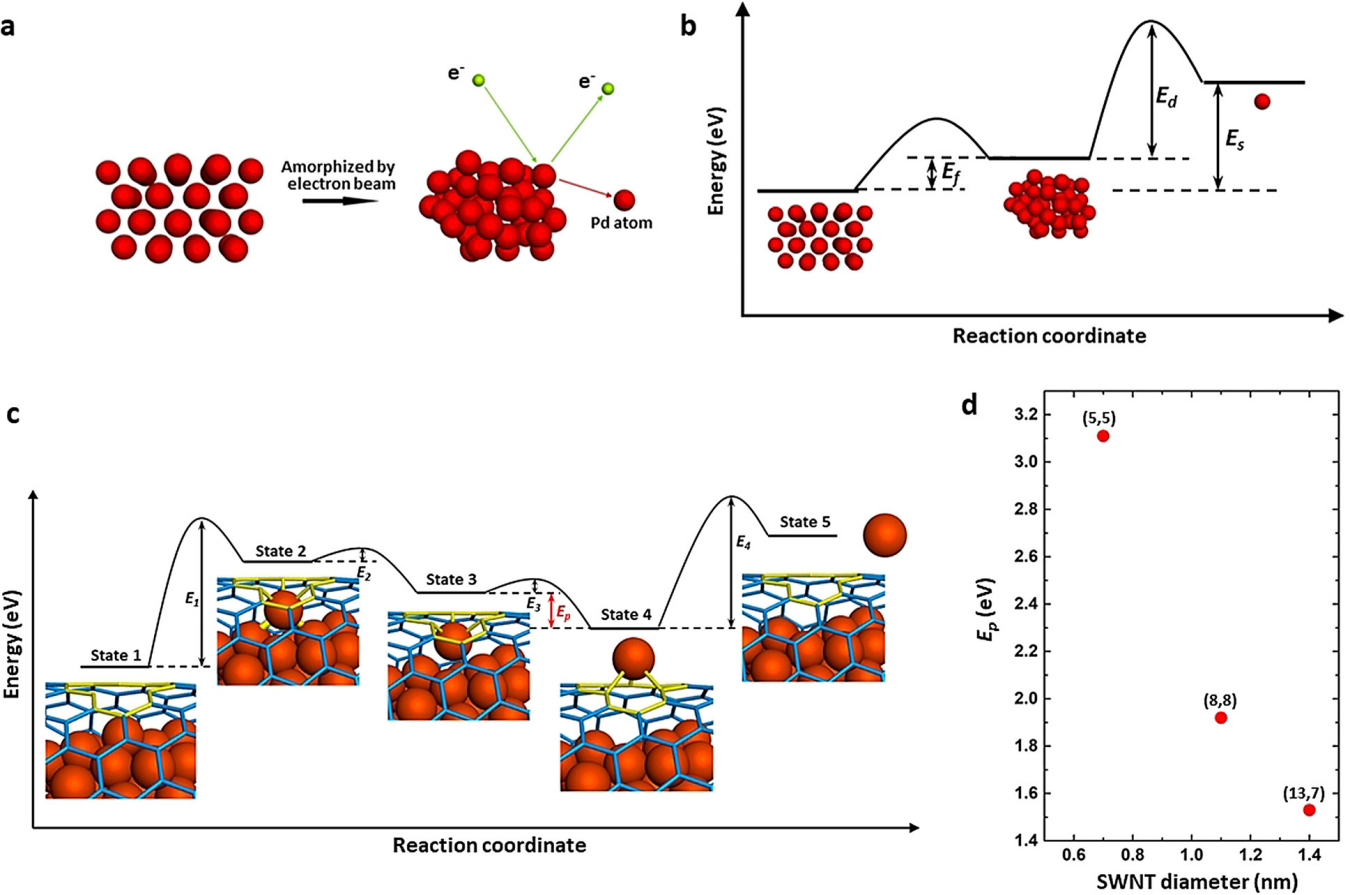
a) Schematic diagram showing detailed processes of a Pd nanocrystal being amorphized and then sputtered by the electron beam. b) Total free energy per atom for Pd nanocrystal, amorphous Pd nanocluster and free Pd atom. c) Schematic energy profile of the process for Pd atom permeation through a monovacancy in the SWNT. State 1, the initial state where an amorphized Pd nanocluster is confined in a (13,7) SWNT with a monovacancy, indicated in yellow. State 2, the Pd atom is activated by incident electrons and bonds to the vacancy. State 3, the outermost Pd atom binding to the concave side of the vacancy is separated from the nanocluster by incident electrons, with intermetallic bonds broken. State 4, the Pd atom moves through the vacancy defect and bonds to the convex side of the SWNT, which is a more stable configuration by energy difference *E*_p_. State 5, the surface‐adsorbed Pd atom is sputtered into vacuum by e‐beam. d) Calculated energy difference *E*_p_ for SWNTs with different chirality and diameters.

The size of the defect is approximately 0.28 nm in profile and is able to accommodate a single Pd atom as shown in Figure S4, which conforms to the features of monovacancy in graphenic lattice although the precise atomic structure of the defect could not be observed. Thus, the monovacancy has been applied for the following theoretical calculations. The whole process appears to follow several stages (Figure 4 c), starting with the amorphization of the crystalline Pd nanoparticle activating the surface atoms (State 1). Next, one of the metal atoms forms Pd−C bonds with the concave side of the nanotube defect stimulated by incident electrons, utilizing the dangling bonds (State 2). As a result of bonding with the defect in the carbon lattice, metallic interactions between the surface Pd atom and the rest of the nanocluster are weakened, making sputtering of the Pd atom by incident electrons easier. After the Pd atom is sputtered, it solely bonds to the vacancy defect, saturating the three dangling bonds (State 3). Thus the Pd atom is trapped by the vacancy in the SWNT, which could be viewed as a substitutional defect of metal atom in the graphenic structure.^[23]^ Due to a significant steric strain when the metal is bonded to the concave surface of the nanotube, with a difference in the bonding energy (*E_p_*),[^18^, ^24^] Pd atom moves through the defect and forms stronger bonds with the convex surface of the nanotube (State 4). The energy barrier (*E_3_*) between State 3 and State 4 is overcome by the transferred kinetic energy from incident electrons to the Pd atom, considering the electron beam is the only stimulus for the whole process. Density functional theory (DFT) simulations have been carried out to calculate *E_p_* for a Pd atom bonded to a monovacancy in SWNTs with different chirality and diameters (Figure 4 d). The mean Pd−C bond lengths and C‐Pd−C bond angles with the Pd atom bonded on the inside (endo‐) and outside (exo‐) of the SWNTs are presented in Table S1. For the (13,7) SWNT in this experiment, with a diameter of 1.4 nm, the endo‐bonding structure has a higher energy than the exo‐bonding structure with an energy difference *E_p_* of 1.53 eV. The *E_p_* for the SWNTs with chirality of (5,5) and (8,8), with diameters 0.7 and 1.1 nm, respectively, have been calculated to be 3.11 and 1.92 eV. This demonstrates that narrower SWNTs with higher curvature have a higher *E_p_*, which could drive the permeation of Pd atoms through the SWNT wall more easily. In addition, a Mulliken population analysis of the charge distribution in the different SWNTs with Pd in the endo‐ and exo‐ configurations indicates that the exo‐configuration allows for a larger charge transfer from Pd atom to SWNT by approximately 0.1 e^−^ (Figure S5). The Pd atom adsorbed on the defect blocks the orifice and hinders the permeation of further Pd atoms which slows down the process, as observed in our experiments (0–37 s and 38–136 s). The electron beam sputters the Pd atom from the surface, thus opening the vacancy defect for the next Pd atom in the final stage of the process (State 5). In some cases, the removed Pd atom does not escape into the vacuum but bonds to the carbon lattice surrounding the monovacancy which would also interact with the next permeated Pd atoms, forming a Pd dimer or few‐atom Pd cluster that temporarily blocks the defect and slows down the permeation process (images of 50 s, 79 s, 85 s and 110 s in Figure 3 b). Thus the permeation of Pd atoms is promoted by the different bonding states between the Pd atom and vacancy, and stimulated by incident electrons (80 keV). The permeation mechanism in this work is different to the contraction force driven migration of metal atoms through carbon onions under the extreme conditions of high energy electron beam irradiation (1250 keV) and high temperature (700 to 1300 K).^[11]^ The observed phenomenon has implications for the permeation process of gas molecule or ions in solution through porous graphene, as graphene is not perfectly flat and may have significant local curvature when pressure difference is applied as a driving force.^[6a]^ Thus, the curvature effect may play a role in many permeation processes through porous graphene, promoting the transport of atoms, ions and molecules from the concave to convex side. In addition, we demonstrate that a metal nanoparticle containing around 120 atoms, much larger than the size of the defect in the nanotube, can permeate across the wall of a SWNT through a sub‐nanometer defect, atom‐by‐atom, despite the fact that the sputtering barrier directly from metal surface is significantly higher than the energy available to Pd atoms in our experiments. The observed amorphization of crystalline Pd nanoparticle appears to be an essential prerequisite for atomic permeation, with the curvature of the carbon lattice playing a key role in promoting the detachment of metal atoms from the nanoparticle and their transport through the defect.

In this study, we directly image the permeation process of Pd atoms across a defect in the carbon lattice of SWNT, atom‐by‐atom, in real time and with atomic resolution. The rate of permeation correlates with the phase state of the metal nanoparticle: the crystalline part of the Pd nanoparticle gradually changing into an amorphous, liquid‐like state provides accessible metal atoms with low coordination number for transport across the lattice defect. This methodology provides the first direct observation of the atomic permeation through a subnano‐pore, highlighting the importance of chemical bonding between the mobile atom and dangling bonds around the subnano‐pore orifice. Curvature of the carbon lattice is shown to drive the atomic permeation in a direction from the concave to convex side of the membrane, due to a difference between metal‐carbon bonding with two opposite sides of the carbon lattice. This new phenomenon and underlying atomistic permeation mechanism are likely to play a role in the filtration processes by porous graphenic carbon‐based membranes, application of which for nano‐filtration has recently become an important area of research.

## Conflict of interest

The authors declare no conflict of interest.

## Supporting information

As a service to our authors and readers, this journal provides supporting information supplied by the authors. Such materials are peer reviewed and may be re‐organized for online delivery, but are not copy‐edited or typeset. Technical support issues arising from supporting information (other than missing files) should be addressed to the authors.

Supplementary

Supplementary
