# Supplementary material for: Direct Imaging of Atomic Permeation Through a Vacancy Defect in the Carbon Lattice
Source: Angew Chem Int Ed Engl. 2020 Oct 25;59(51):22922–7. doi: 10.1002/anie.202010630 (PMC7814674; doi:10.1002/anie.202010630)
Supplement: Supplementary file 1 — Supplementary [file ANIE-59-22922-s001.pdf]

Angewandte Chemie
Eine Zeitschrift der Gesellschaft Deutscher Chemiker

Supporting Information

Direct Imaging of Atomic Permeation Through a Vacancy Defect in the Carbon Lattice

Kecheng Cao[+], Stephen T. Skowron[+], Craig T. Stoppiello, Johannes Biskupek, Andrei N. Khlobystov, and Ute Kaiser**

anie_202010630_sm_miscellaneous_information.pdf
anie_202010630_sm_Movie_S1.mp4

Table of Contents

1. Experimental Procedures
2. Results and Discussion

1. Experimental Procedures

Arc-discharge SWNTs (Carbon Solutions, USA) were thermally treated to open the termini and to remove the residual amorphous carbon from the surface. The as-received SWNTs were heated in air for 30 min at 600 °C. The freshly opened SWNTs were mixed with a twofold by weight excess of palladium hexafluoroacetylacetonate ($Pd(C_5HF_6O_2)_2$) compound (Sigma-Aldrich). The resultant mixture was then sealed in a Pyrex glass tube under reduced pressure (10^{-6} mbar) and heated to 140 °C for 3 days. The sample was then rapidly cooled to room temperature, and the SWNTs were washed with tetrahydrofuran (100 ml) to remove any species from the outside of the nanotube and filtered through a polytetrafluoroethylene membrane (pore size = 0.2 μm). The filled SWNTs were heated at 500°C for 3 hours in a Pyrex tube under argon to decompose the $Pd(C_5HF_6O_2)_2$ molecules. The sample was then dispersed in isopropanol and drop-cast onto a lacey carbon-coated copper TEM grid for HRTEM analysis. Time-series AC-HRTEM images were acquired on an image-side C_s-corrected FEI Titan 80-300 TEM operated at 80 kV at room temperature. The TEM specimen was heated in air at 150 °C for 5 min shortly before insertion into the TEM column. The electron flux applied to the samples was 4.3×10^6 $e^-/nm^2\cdot s$. Images were acquired using a Gatan Ultrascan 1000XP CCD and exposure times between 0.5 to 1.0 s.

DFT calculations were performed using the Q-Chem 5.0 quantum chemistry software package[1] with the BLYP exchange-correlation functional[2], a 6-31G basis set for C and H atoms, and the SRSC effective core potential for Pd atoms[3]. The SCF convergence criterion was 10^{-8}, and the threshold for neglect of two electron integrals was 10^{-12} E_h. Geometry optimizations were performed for the six structures shown in Supplementary Tab. 1: endo- and exo-configurations of a (5,5) SWNT $PdC_{100}H_{20}$, (8,8) SWNT $PdC_{160}H_{32}$, and (13,7) SWNT $PdC_{412}H_{40}$; for singlet and triplet electronic states. In agreement with previous studies[4], the singlet was found to be the lower energy ground state in all cases, and all reported values correspond to this multiplicity.

2. Results and Discussion

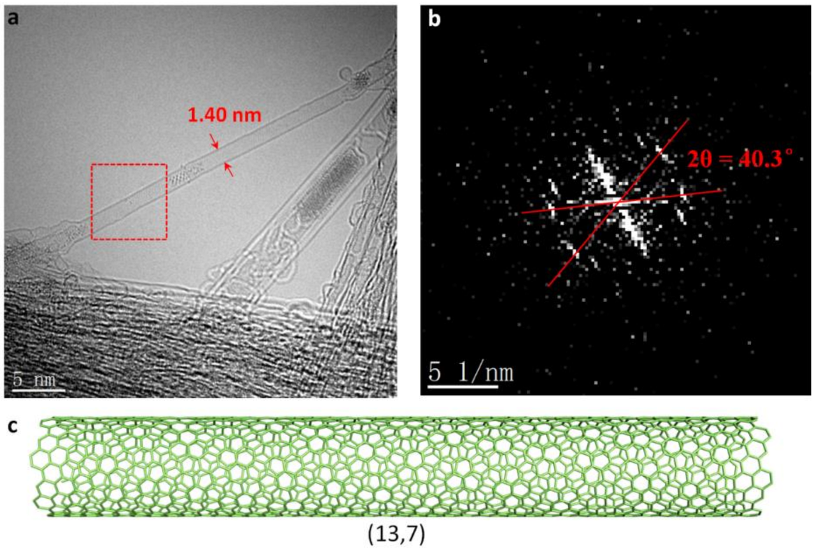

Figure S1. Determination of the chiral index of the SWNT in Figure 1. (a) Diameter of the SWNT is measured in a raw AC-HRTEM image from Supplementary Movie S1. (b) Fast Fourier transform pattern of the area boxed in (a). (c) Structural model of the SWNT in (a) with determined chiral index of (n=13; m=7).

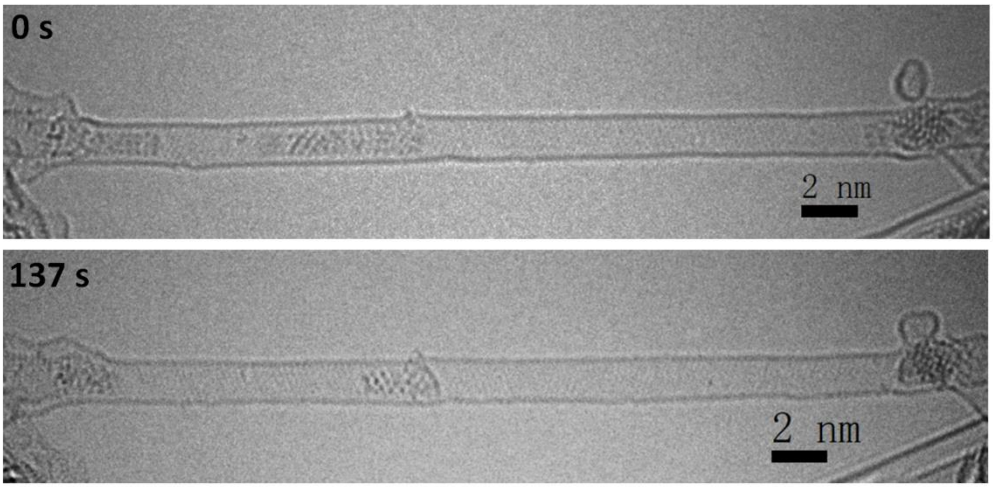

Figure S2. Raw AC-HRTEM image showing the initial state (0 s) and late state (137 s) of the Pd nanoparticle confined in the SWNT with both ends remaining blocked.

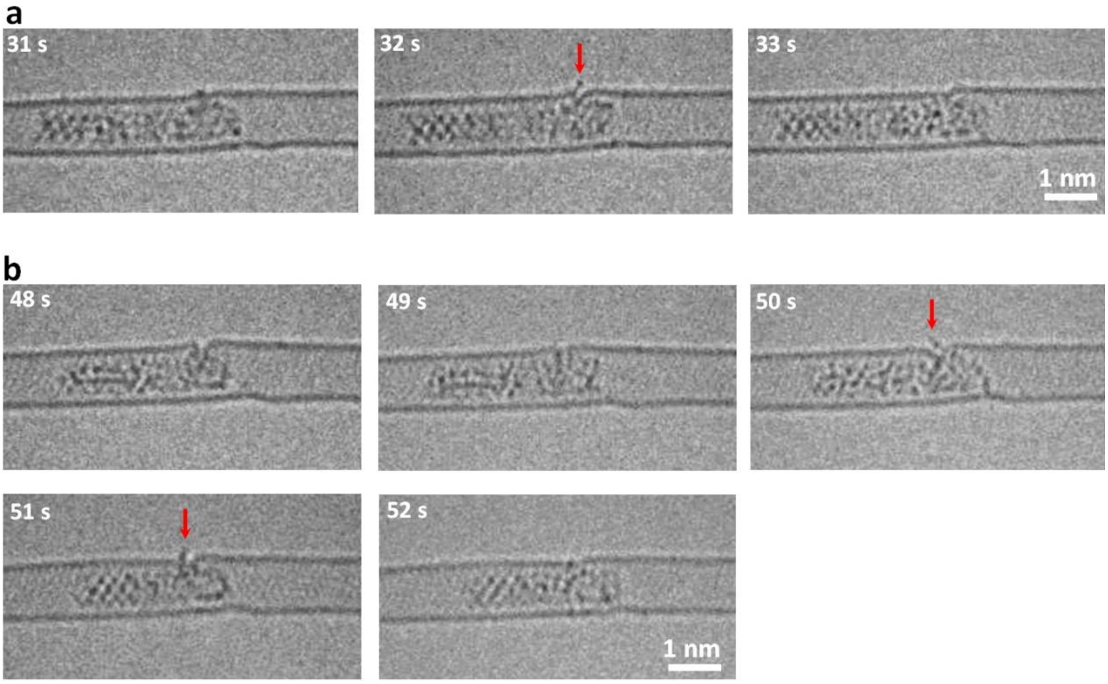

Figure S3. Two time series AC-HRTEM images showing the detailed permeation processes. The permeated Pd atoms bonding to the vacancy from outside are indicated with red arrows.

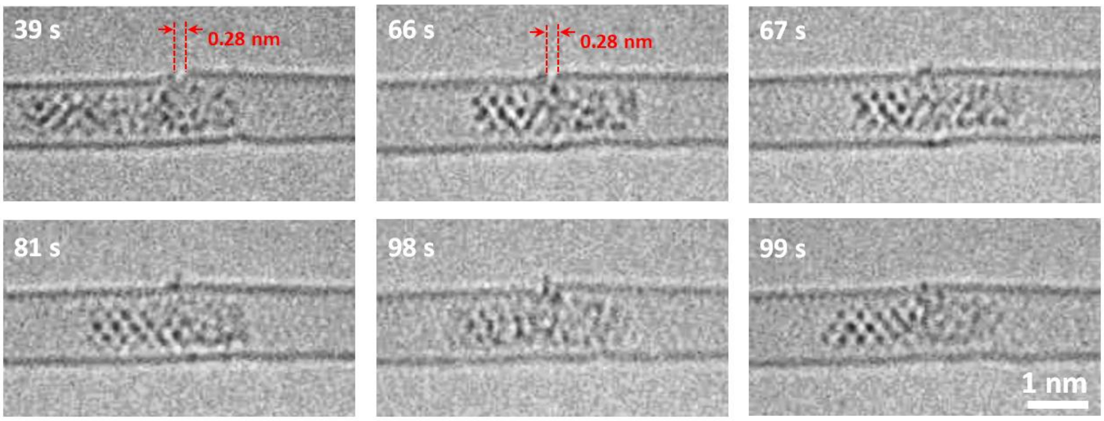

Figure S4. Time series AC-HRTEM images showing the different state of the defect. At 39 s and 66 s, the defect is open with size of 0.28 nm. At 67 s and 99 s, single Pd atom is trapped in the defect. At 81 s and 98 s, two Pd atoms 'stand' on the defect.

Table S1. Atomic models for the calculations of Pd doped SWNTs and calculated mean Pd-C bond length, mean C-Pd-C bond angle and energy difference.

Chirality	endo-configuration		exo-configuration		Diameter	Mean Pd-C bond length	Mean C-Pd-C bond angle	Energy difference (endo-exo)
	top view	side view	top view	side view				
(5,5)	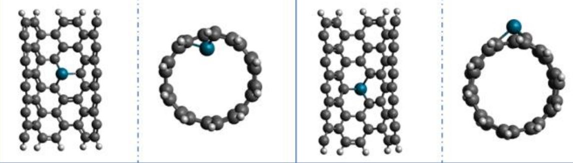				0.7 nm	endo: 0.197 nm<br>exo: 0.199 nm	endo: 95.8°<br>exo: 91.3°	3.11 eV
(8,8)	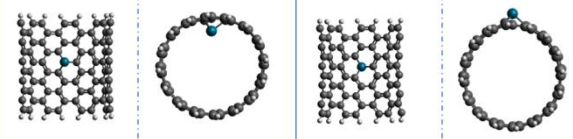				1.1 nm	endo: 0.198 nm<br>exo: 0.199 nm	endo: 86.8°<br>exo: 89.7°	1.92 eV
(13,7) Experimental SWNT	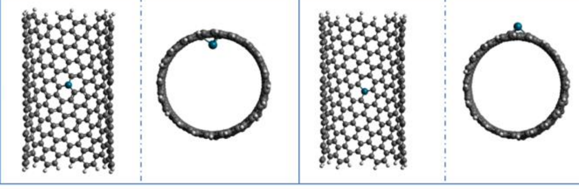				1.4 nm	endo: 0.198 nm<br>exo: 0.198 nm	endo: 89.5°<br>exo: 86.3°	1.53 eV

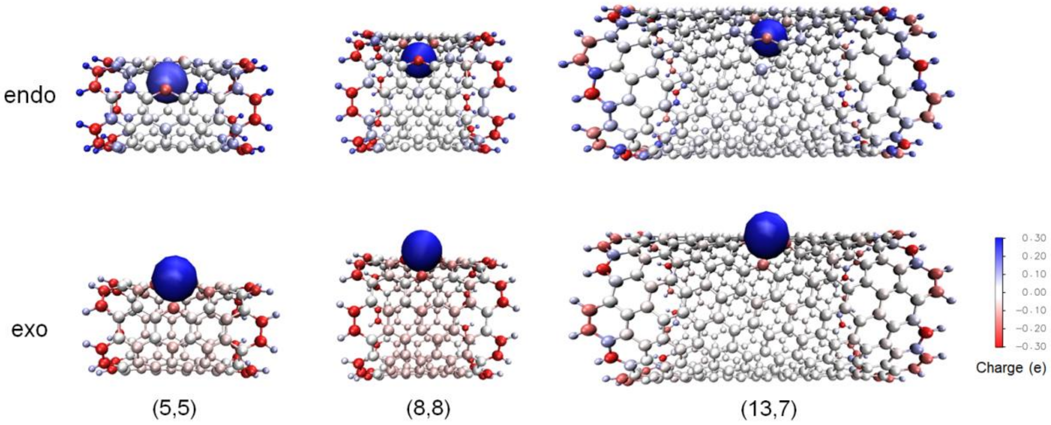

Figure S5. Charge distribution of Pd doped SWNTs by Mulliken population analysis. The partial charges of Pd: SWNT (5,5) endo 0.08 e, exo 0.22 e with difference of 0.14 e; SWNT (8,8) endo 0.15 e, exo 0.26 e with difference of 0.11 e; SWNT (13,7) endo 0.18 e, exo 0.28 e with difference of 0.10 e.

References

[1] Y. Shao, Z. Gan, E. Epifanovsky, A. T. B. Gilbert, M. Wormit, J. Kussmann, A. W. Lange, A. Behn, J. Deng, X. Feng, D. Ghosh, M. Goldey, P. R. Horn, L. D. Jacobson, I. Kaliman, R. Z. Khaliullin, T. Kuś, A. Landau, J. Liu, E. I. Proynov, Y. M. Rhee, R. M. Richard, M. A. Rohrdanz, R. P. Steele, E. J. Sundstrom, H. L. Woodcock, P. M. Zimmerman, D. Zuev, B. Albrecht, E. Alguire, B. Austin, G. J. O. Beran, Y. A. Bernard, E. Berquist, K. Brandhorst, K. B. Bravaya, S. T. Brown, D. Casanova, C.-M. Chang, Y. Chen, S. H. Chien, K. D. Closser, D. L. Crittenden, M. Diedenhofen, R. A. DiStasio, H. Do, A. D. Dutoi, R. G. Edgar, S. Fatehi, L. Fusti-Molnar, A. Ghysels, A. Golubeva-Zadorozhnaya, J. Gomes, M. W. D. Hanson-Heine, P. H. P. Harbach, A. W. Hauser, E. G. Hohenstein, Z. C. Holden, T.-C. Jagau, H. Ji, B. Kaduk, K. Khistyaev, J. Kim, J. Kim, R. A. King, P. Klunzinger, D. Kosenkov, T. Kowalczyk, C. M. Krauter, K. U. Lao, A. D. Laurent, K. V. Lawler, S. V. Levchenko, C. Y. Lin, F. Liu, E. Livshits, R. C. Lochan, A. Luenser, P. Manohar, S. F. Manzer, S.-P. Mao, N. Mardirossian, A. V. Marenich, S. A. Maurer, N. J. Mayhall, E. Neuscamman, C. M. Oana, R. Olivares-Amaya, D. P. O'Neill, J. A. Parkhill, T. M. Perrine, R. Peverati, A. Prociuk, D. R. Rehn, E. Rosta, N. J. Russ, S. M. Sharada, S. Sharma, D. W. Small, A. Sodt, et al., *Molecular Physics* **2014**, *113*, 184-215.

[2] a)A. D. Becke, *Phys Rev A Gen Phys* **1988**, *38*, 3098-3100; b)C. Lee, W. Yang, R. G. Parr, *Phys Rev B Condens Matter* **1988**, *37*, 785-789.

[3] D. Andrae, U. Huermann, M. Dolg, H. Stoll, H. Preu, *Theoretica Chimica Acta* **1990**, *77*, 123-141.

[4] Y. K. Chen, L. V. Liu, W. Q. Tian, Y. A. Wang, *The Journal of Physical Chemistry C* **2011**, *115*, 9306-9311.

Author Contributions

C. T. S. prepared the sample. K. C. investigated of the sample by AC-HRTEM and recorded the videos of permeation. K. C., S. T. S., J. B., A. N. K. and U. K. discussed the results and analyzed the data. S. T. S. carried out theoretical modelling. K. C., S. T. S, J. B., A. N. K. and U. K. drafted the manuscript. All the authors have revised the manuscript. U. K. and A. N. K. supervised the research.
